# Multiplex Microarrays in 96-Well Plates Photoactivated with 4-Azidotetrafluorobenzaldehyde for the Identification and Quantification of β-Lactamase Genes and Their RNA Transcripts

**DOI:** 10.3390/cimb46010005

**Published:** 2023-12-20

**Authors:** Mariya M. Ulyashova, Galina V. Presnova, Anna A. Filippova, Vitaly G. Grigorenko, Alexey M. Egorov, Maya Yu. Rubtsova

**Affiliations:** Department of Chemistry, Lomonosov Moscow State University, 119991 Moscow, Russia; mmu@enzyme.chem.msu.ru (M.M.U.); gkovba@enzyme.msu.ru (G.V.P.); iiffii@mail.ru (A.A.F.); GrigorenkoVG@my.msu.ru (V.G.G.); aegorov@enz.chem.msu.ru (A.M.E.)

**Keywords:** antibiotic-resistant bacteria, molecular detection, oligonucleotide microarrays, β-lactamases, photoactivation, bifunctional linkers

## Abstract

Antibiotic-resistant bacteria represent a global issue that calls for novel approaches to diagnosis and treatment. Given the variety of genetic factors that determine resistance, multiplex methods hold promise in this area. We developed a novel method to covalently attach oligonucleotide probes to the wells of polystyrene plates using photoactivation with 4-azidotetrafluorobenzaldehyde. Then, it was used to develop the technique of microarrays in the wells. It consists of the following steps: activating polystyrene, hybridizing the probes with biotinylated target DNA, and developing the result using a streptavidin–peroxidase conjugate with colorimetric detection. The first microarray was designed to identify 11 different gene types and 16 single-nucleotide polymorphisms (SNPs) of clinically relevant ESBLs and carbapenemases, which confer Gram-negative bacteria resistance to β-lactam antibiotics. The detection of *bla* genes in 65 clinical isolates of *Enterobacteriaceae* demonstrated the high sensitivity and reproducibility of the technique. The highly reproducible spot staining of colorimetric microarrays allowed us to design a second microarray that was intended to quantify four different types of *bla* mRNAs in order to ascertain their expressions. The combination of reliable performance, high throughput in standard 96-well plates, and inexpensive colorimetric detection makes the microarrays suitable for routine clinical application and for the study of multi-drug resistant bacteria.

## 1. Introduction

Antibiotic-resistant bacterial infections represent a serious global threat [1,2]. In order to improve infection control and realize a timely choice of chemotherapy tactics, the early identification of bacterial resistance is required. The variety of mechanisms of bacterial resistance to antibiotics and the factors that implement them necessitate the development of molecular multiplex analysis technologies with high productivity [3].

The production of bacterial β-lactamases is the main mechanism of resistance of Gram-negative pathogens to β-lactam antibiotics [4]. β-Lactamases are diverse in structure and substrate specificity with respect to different groups of β-lactams. A peculiarity of multi-drug resistant bacteria is the localization of several β-lactamase genes onto one plasmid [5]. The development of technologies that enable the simultaneous determination of carbapenemase and extended-spectrum β-lactamase (ESBL) genes is extremely relevant for the detection of resistance to β-lactams [6,7,8]. 

High multiplicity and throughput are the distinctive features of DNA microarray technology, and it has huge research and diagnostic potential in this field [9]. The identification of DNA by hybridization with specific oligonucleotide probes immobilized on solid supports, which was first proposed by Wallace in 1979 [10], has found wider applications after the development of a miniaturized platform based on microarrays with hundreds or thousands of oligonucleotides immobilized in micron-diameter spots. A large variety of surfaces have been used as supports for DNA microarrays, including silicon [11], polymeric substrates such as poly(methylmethacrylate), polycarbonate, polystyrene, and porous membranes (nylon and nitrocellulose) [12,13], but the most popular is glass slides [14]. So far, several DNA microarray platforms have been developed for antibiotic resistance gene identification [15,16,17]. Various designs of microarrays have been proposed for the identification of β-lactamase genes [18,19]. 

Nevertheless, the volume of routine use of DNA microarrays is not large and does not correspond to the potential of this analytical tool. The main factors that prevent their widespread application are the high labor intensity as well as the sufficiently high cost of materials and equipment for the analysis. Microarrays that have the potential for automation or represent a part of “Point of Care” devices greatly simplify their use in clinical laboratories. Using technological solutions that combine several microarrays onto one holder make it easier to carry out the stages of incubation, washing, and detection. The most promising approach is the placement of microarrays onto the wells of microtiter plates that have shown high performance in the field of immunoassays. Microtiter plates have the potential to expand the scope and adapt this multiplex technology for low-resource laboratories.

Surface chemistry is essential to preserve the capture probes’ functionality and boost their effectiveness [20]. In this work, we used various bifunctional linkers (4-azidobenzoic acid (ABA), its N-hydroxysuccinimide ester (HEABA), and 4-azidotetrafluorobenzaldehyde (ATFB)) to chemically modify conventional 96-well polystyrene plates using a photoactivation technique. The most efficient method (modification with ATFB) was then applied to fabricate DNA microarrays for the purposes of identifying clinically relevant β-lactamase genes and quantifying their RNA transcripts. Two designs of microarrays in the wells were developed. The first was for the identification of eight different types of carbapenemase genes of classes A, B, and D (*bla*_KPC_, *bla*_NDM_, *bla*_VIM_*, bla*_IMP_, *bla*_SPM_, *bla*_GIM_*, bla*_SIM_, and *bla*_OXA_), three different types of ESBL genes (*bla*_TEM_, *bla*_SHV_, and *bla*_CTX-M_), and sixteen single-nucleotide polymorphisms (SNPs) in the *bla* genes of class A. The purpose of the second microarray was to quantify the mRNAs of four different *bla* gene types: *bla*_TEM_, *bla*_CTX-M-1_, *bla*_NDM_, and *bla*_OXA-48_. The colorimetric microarrays in the wells showed the effective multiplex identification of ESBL and carbapenemase genes and the potential for the quantitative determination of *bla* mRNAs isolated from cell cultures.

## 2. Materials and Methods

### 2.1. Reagents

dNTPs, Taq DNA polymerase, DNase I, and dUTP-11-Biotin were obtained from Fermentas (St. Leon-Rot, Germany). N-ethyl-N-(3-dimethylaminopropyl)carbodiimide hydrochloride (EDC), N-hydroxysuccinimide (NHS), sodium dodecyl sulfate (SDS), bovine serum albumin (BSA), 3,3,5,5-tetramethylbenzidine dihydrochloride (TMB), casein, ethylene glycol, Triton X-100, Tween-20, and dextran sulfate sodium salt were purchased from Sigma (St. Louis, MO, USA). Streptavidin-HRP conjugate was obtained from Imtek (Moscow, Russia). The 4-azidotetrafluorobenzaldehyde (ATFB), 4-azidobenzoic acid (ABA), and N-hydroxysuccinimide ester of 4-azidobenzoic acid (HEABA) were synthesized accordingly [21,22]. The 96-well polystyrene microtiter plates were purchased from Greiner Bio-One, (Frickenhausen, Germany). Acids, alkali, salts, and solvents were purchased from Chimmed (Moscow, Russia). All chemicals and organic solvents were of analytical grade. Water used in all experiments was purified with a Milli-Q system (Millipore, Billerica, MA, USA).

Primers and oligonucleotide probes containing a 5′-aminolink and 13-mer thymidine spacer were synthesized and purified by Syntol (Moscow, Russia). The structures of the probes for the identification of β-lactamases and carbapenemase genes, including those for the identification of the key SNPs, and the primers for multiplex PCR were described earlier [23,24]. Sequence of model oligonucleotide HC containing a 5′-biotin was complementary to the control oligonucleotide probe PHC of 19 bases.

### 2.2. Bacterial Strains and DNA and RNA Samples

Collections of bacterial strains and DNA fractions isolated from control and clinical strains were used for the development of microarrays: (a) laboratory strains of *E. coli,* producers of recombinant β-lactamases; (b) a collection of DNA isolated from control strains resistant to β-lactam antibiotics (*E. coli* J53 (TEM-1), *E. coli* J53.2 (TEM-3), *E. coli* J53.2-pUD16 199 (TEM-37 or IRT 8), *K. pneumoniae* CTA-1 (SHV-1), CTA-3 (SHV-5), CTA-5 (SHV-5), *E. coli* MSP493 (CTX-M-9), *E. coli* RESORT-2320 (CTX-M-42), *K. pneumoniae* SP477.1 (CTX-M-3), *E.coli* BH3223/2 (CTX-M-15), *E. coli* RESORT-333 (CTX-M-14), *K. pneumonia* AO8053 (KPC-3)*, P. aeruginosa* VR-143/97 (VIM-1), *P. aeruginosa* RESORT 565 (VIM-2), *P. aeruginosa* 101/1477 (IMP-1), *A. baumannii* AC-54/97 (IMP-2), *P. aeruginosa* 48-28 (SPM-1), *A. baumannii* KARAT-44 (OXA-58), *A. baumannii* KARAT-30 (OXA-24), *A. baumannii* RESORT 547 (OXA-23), clinical strains *K. pneumoniae* KPB417/16 (SHV-11 CTX-M-15, TEM-1, NDM-1, OXA-48) *K. pneumonia* KPB882/14 (CTX-M-15, TEM-1, NDM-1, OXA-48); and a collection of clinical strains (Table S1). Phenotypic characterization was performed using the disk-diffusion method, and genotyping was carried out by real-time PCR and partial sequencing. DNA fractions isolated from control and clinical strains were kindly provided by Dr. M.V. Edelstein (Institute of Antimicrobial Chemotherapy of Smolensk State Medical Academy, Smolensk, Russia) and Dr. N.K. Fursova (State Research Center for Applied Microbiology & Biotechnology, Obolensk, Russia). DNA fractions were obtained with the InstaGene Matrix Kit (Bio-Rad, Hercules, CA, USA) and used as templates for multiplex PCR amplification.

The fractions of total RNA were isolated from laboratory strains of *E. coli,* producers of recombinant β-lactamases, using the RNA-Extran kit (Syntol, Moscow, Russia) according to a protocol of the manufacturer.

β-lactamase mRNA standards were obtained by transcription in vitro: plasmid DNA was isolated from the *E. coli*-producing TEM-1, CTX-M-116, OXA-48, and NDM-1 using Plasmid Midiprep 2.0 kit. Then, the fragments of the pET24 plasmid including full-size β-lactamase genes and T7 promotor were amplified using direct (5′-TCCGGATATAGTTCCTCCTTTCA-3′) and reverse (5′-AGATCTCGATCCCGCGAA-3′) primers. Synthesis of specific mRNAs was carried out using a T7-Transcription kit (Biolabmix, Novosibirsk, Russia); 1 mL of PCR product was mixed in a volume of 50 mL with a transcription buffer, a mixture of dNTPs at a final concentration of 1 mM, and T7 RNA polymerase (3 e.a./mL), incubated for 2 h at 37 °C. Residual amounts of DNA were removed using DNase (2 e.a./mL, 37 °C, 30 min). The obtained RNA samples were additionally purified on “QIAquick” columns according to the manufacturer’s protocol. The cDNA strands were obtained as follows: 2 µL of a total RNA sample and primers (Random (dNTP)_10_) at a final concentration of 0.5 µm were annealed (70 °C, 2 min) and then cooled on ice; buffer (250 mM Tris–HCl, 250 mM KCl, 20 mM MgCl_2_, pH 8.3), a mixture of dNTPs at a final concentration of 1 mM, and 1 µL of MMLV revertase (Evrogen, Moscow, Russia) were added. The mixture was incubated at 42 °C for 1 h, and the reaction was stopped by heating (70 °C, 10 min). Samples of β-lactamase mRNAs were used as templates for multiplex PCR amplification.

### 2.3. Photochemical Activation of Polystyrene Surface

Photochemical activation of polystyrene microtiter plates was carried out using solutions of ABA (50 mM in methanol:DMFA (75:25)), HEABA (50 mM in methanol:DMFA (75:25)), and ATFB (50 mM in methanol). Aliquots of 20 µL were poured into each well of the plate and allowed to air dry in the dark. After complete evaporation of solvent, the photolinker-coated plate was exposed to UV light of 365 nm in a UV transilluminator (Model 2400; Stratagene, La Jolla, CA, USA). After irradiating, the plates were washed twice with methanol. When using ABA, additional activation of carboxylic groups with EDC was performed; the wells were acidified by rinsing with 0.1 M HCl (5 min), and then incubated in a solution containing 1% EDC and 0.2% NHS at room temperature for 30 min, followed by rinsing with distilled water for 1 min.

### 2.4. Oligonucleotide Microarray Fabrication

The oligonucleotide probes (20 μM in a mixture of 160 mM Na_2_SO_4_ and 130 mM Na_2_HPO_4_) were spotted onto the wells of microtiter plates (or epoxy-coated glass slides as a control) with XactII™ Microarrayer (Lab Next Inc., Glenview, IL, USA). Then, the wells were heated at 60 °C for 30 min and washed as follows: (1) 0.1% Triton X-100 in water at RT for 5 min; (2) 0.05% HCl in water at RT for 2 min; (3) 25 mM KCl in PBS at 50 °C for 15 min and blocked in a solution of 1% BSA and 1% casein in PBS for 30 min at 37 °C. The epoxy-coated glass slides were rinsed and blocked according to manufacturer guidelines.

### 2.5. Microarray Hybridization and Detection

Target DNAs of β-lactamases were amplified by means of multiplex PCR using a mixture of primer pairs and labeled with biotin by introducing dUTP-11-biotin, as described in [24]. Biotinylated PCR products were fragmented with DNase I (0.5 mU per 1 ng of DNA) at RT for 5 min to obtain sizes of about 50 to 150 bp. Then, 250–750 ng of fragmented DNA targets were resuspended in 60 µL of 2× SSPE buffer (0.2 M NaH_2_PO_4_, 3.0 M NaCl, 20 mM EDTA, 0.1% SDS pH 7.4) containing 1 nM biotinylated control oligonucleotide as the target for positive hybridization control, added to the wells of microtiter plates, and incubated at 45 °C for 1.0 h in a Thermomixer Comfort (Eppendorf AG, Hamburg, Germany) with shaking at 600 rpm. After the hybridization, the plate wells were washed three times with PBS at RT for 2 min with shaking at 600 rpm.

For the detection, the wells were incubated with a solution of streptavidin-HRP conjugate (1 µg/mL) in PBST for 30 min at 37 °C, and then washed three times with PBS at RT for 2 min, and incubated in a substrate solution (5 mM TMB, 1 mM H_2_O_2_, 0,5% dextrane sulfate) at RT for 10 min. After washing with distilled H_2_O and drying, the microarrays were scanned with the flatbed scanner Perfection V750 Pro (Epson) with a resolution of 4800 dpi. The software package ScanArray Express Version 3.0 was used for image analysis of the TIFF files generated by the scanner. Background-corrected signals were collected for each individual spot and then processed with Microsoft Excel 2010 (Version 15.0). The limit of detection (LOD) of target oligonucleotide or target DNA was calculated as the mean signal intensity registered for a blank sample (0 pM DNA) plus two standard deviations (SD; *n* = 10).

## 3. Results

### 3.1. Photoactivation of 96-Well Polystyrene Plates with Different Bifunctional Linkers

The use of photolinkers (photoactivatable bifunctional linkers) represents an effective technique for attaching biomolecules to polymer surfaces [25], and aryl azides are often used for modification as photolinkers [26,27]. Upon photolysis, phenyl azide groups form short-lived nitrenes that react rapidly with the surrounding chemical environment. The intermediates of aryl azides can undergo ring expansion to create dehydroazepines that have a tendency to react preferentially with nucleophiles, especially amines.

**Figure 1:**
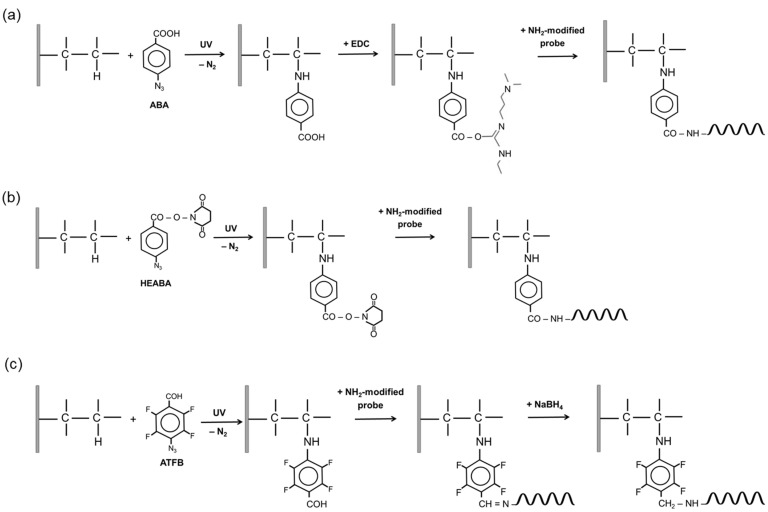
Schemes for photomodification of polystyrene with bifunctional linkers: (**a**) 4-azidobenzoic acid (ABA); (**b**) N-hydroxysuccinimide ester of 4-azidobenzoic acid (HEABA); (**c**) 4-azidotetrafluorobenzaldehyde (ATFB).

In this study, we compared the effectiveness of three bifunctional photolinkers based on aryl azides (ABA, HEABA, and ATFB) for the chemical modification of polystyrene to provide a covalent immobilization of oligonucleotide probes. The schemes for polystyrene surface modification are presented in Figure 1. The principle of photochemical activation consists of the following: the azido group of a bifunctional linker forms a highly reactive nitrene intermediate in the presence of UV light, which inserts into the C-H bond of polystyrene. The other functional group of the photolinker (carboxylic, ester, or aldehyde) remains intact and further reacts with the amino group of the oligonucleotide, forming a covalent bond. When using ABA, the additional activation of carboxylic groups with EDC/NHC was performed. In order to optimize the photoactivation procedure for ensuring the covalent immobilization of oligonucleotides, we used an 18-base control oligonucleotide probe (SC) bearing a 13-mer thymidine spacer with an amino group at the 5′-end and biotin at the 3′–end (5′-NH_2_-TTTTTTTTTTTTT-TCTAGACAGCCACTCATA-biotin). A probe SC without an amino group at the 5′–end was used to check for any non-specific binding, as nucleophilic amino groups in purine and pyrimidine bases can compete with it for binding to the activated surface. After spotting and washing, biotin was revealed with a streptavidin-HRP conjugate and the subsequent colorimetric detection of HRP activity. As a result of the enzymatic reaction, colored spots were formed on the surface. For the evaluation of the results, the spots were scanned to determine an integrated dyeing intensity of each spot that is proportional to the concentration of oligonucleotide probes immobilized on its surface (surface loading).

**Figure 2:**
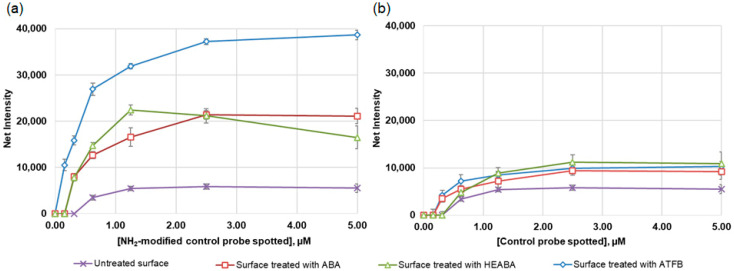
Effect of the control biotinylated probe SC concentration on the intensity of spot staining (mean values of 6 replicate spots) after interaction with a conjugate streptavidin-HRP. The probe was spotted on polystyrene treated with different photolinkers and untreated polystyrene. (**a**) The probe with amino-modification at the 5′-end; (**b**) the probe without amino-modification.

The results demonstrate the differences in the overall intensities of the spots on polystyrene modified with different photolinkers (Figure 2). A significant increase in the amount of SC immobilized on photoactivated polystyrene compared to the untreated one was revealed for all three linkers tested. The results of the immobilization of SC without amino groups were insignificant and comparable on both the treated and nontreated surfaces (Figure 2a). This indicates the specificity of the photolinker-activated polystyrene towards the end-labeled amino group of oligonucleotide probes.

ATFB showed the highest photolinking potential; the signals after treatment with ATFB were 2–2.5 times higher for all SC concentrations compared to the treatment with ABA and HEABA. This result is likely explained by the higher reactivity of aldehyde groups against the amino-modified oligonucleotides compared to the carboxyl group, even after its activation with EDC/NHS. In addition, the presence of four fluorine atoms in the benzene ring of ATFB additionally increases the electrophilicity of the aldehyde groups and stabilizes the nitrene intermediate. These data correlate with those showing that azides with a perfluorinated aryl structure are quite efficient at forming the desired nitrene intermediate [28].

**Figure 3:**
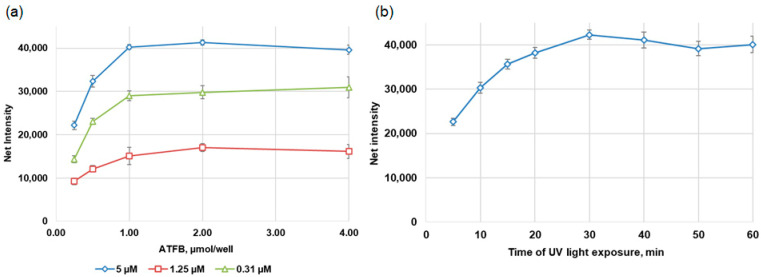
(**a**) Effect of 4-azidotetrafluorobenzaldehyde (ATFB) concentration on the intensity of spot staining (mean values of 6 replicate spots) for different concentrations of the control oligonucleotide probe SC spotted onto polystyrene. (**b**) Dependence of the intensity of staining of the spot with an immobilized control probe SC (mean values of 6 replicate spots) on the time of UV light exposure after treating polystyrene with ATFB (1 µmol/well).

Further, the concentration of the photolinker and the time of irradiation of the 96-well polystyrene plate with UV light were optimized (Figure 3). The most effective activation of polystyrene by ATFB was observed at a concentration of 1 µmol/well (Figure 3a). The results were very similar for all SC concentrations examined. To determine the optimum time for the photoactivation reaction, the ATFB-coated plates (1 µmol/well) were exposed to UV light for 5–60 min (Figure 3b). The highest intensity of staining was achieved by irradiating polystyrene for 30–40 min. However, at the end of the reaction, the polystyrene surface became a light yellow. Therefore, a UV exposure time of 20 min was found to be more optimal because no change in the physical properties of plates, such as the color or shape, was observed during this period. Thus, the whole activation of a 96-well polystyrene plate with ATFB requires no more than 35 min, taking about 10 min for surface coating and solvent evaporation, 20 min for UV exposure, and 4 min for washing.

The stability of the chemically modified surfaces is an important factor for their practical use. To examine the stability, photoactivated polystyrene plates were placed in vacuum-sealed packages and kept at room temperature for 12 months. During this period, the control probe SC was immobilized at 1-month intervals. The immobilization efficiency was almost the same throughout the entire period, and it did not differ from the immobilization of the probe using activation with freshly prepared reagents.

### 3.2. Sensitivity of Hybridization Analysis on Photoactivated 96-Well Polystyrene Plates

The covalent immobilization of amino-modified oligonucleotide probes onto the photoactivated polystyrene surface was further investigated through hybridization efficacy. For this purpose, various amounts of a model oligonucleotide probe of 19 bases (HC) labeled with biotin (5′-biotin-GCTCCTGACTCGTCCAATC-3′) were hybridized with the complementary oligonucleotide probe PHC (5′-NH_2_-TTTTTTTTTTTTTGATTGGACGAGTCAGGAGC-3′) immobilized on photoactivated polystyrene. A special pre-hybridization washing procedure consisting of successive incubations with solutions of Triton X-100, HCl, and KCl was developed to reduce the background staining of the wells. The results of hybridization performed at 45 °C were characterized by the staining of maximum intensity after 1 h of incubation, beyond which no appreciable increase occurred. We compared the sensitivity of hybridization analysis on activated 96-well polystyrene plates and epoxy-modified glass slides. The LODs of the model oligonucleotide probe on these supports were rather similar and corresponded to 0.030 ± 0.010 nM for the 96-well polystyrene plates and 0.020 ± 0.005 nM for the epoxy-modified glass slides. Thus, the sensitivity of the hybridization analysis on 96-well polystyrene plates was quite comparable to that on the epoxy-modified glass slides, which are widely used in microarray technology.

### 3.3. Microarrays in the Wells of the Microtiter Plate for the Identification of ESBLs and Carbapenemases

**Figure 4:**
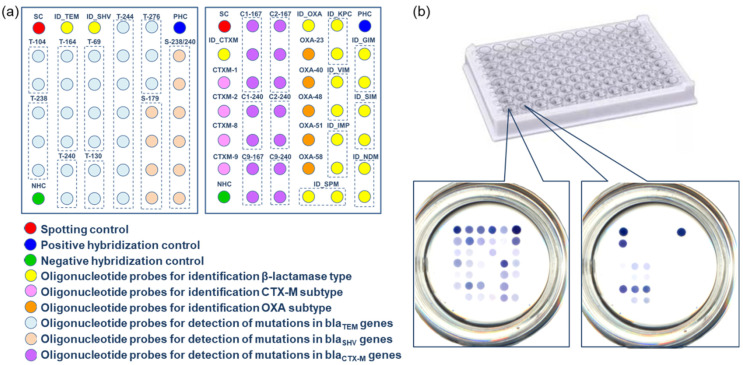
Microarray layout. (**a**) Schematic representation of the capture probes on the microarray. The probe sets are named after the position of the amino acid substitution within the *bla*_TEM_ and *bla*_SHV_ sequences. (**b**) Colorimetric image after hybridization of 750 ng target DNA containing *_bla_*_TEM-1_, *bla*_SHV-1_, and *bla*_CTX-M-9_ at 45 °C for 1.0 h.

We initially utilized the technology of polystyrene modification to develop oligonucleotide microarrays for the purpose of identifying the genes of the most clinically relevant β-lactamases, which include ESBLs, inhibitor-resistant β-lactamases (IRT) of class A, and carbapenemases of classes A, B, and D. The ability to use microarrays with different specificities in separate wells is one benefit of using 96-well plates. The first microarray was designed in the format of two parts, each with dimensions of six by seven spots (42 spots in total in each part, Figure 4a). The probes were arranged in adjacent wells of eight-well strips. Each well contained thirty-nine probes for the identification of the *bla* genes and three control probes, which included a spotting control and positive and negative hybridization controls (Figure 4b). The first well included probes for the identification of *bla*_TEM_ and *bla*_SHV_ of class A and their key mutations (eight SNP positions in *bla*_TEM_, and three SNP positions in *bla*_SHV_). The second well included probes for the identification of *bla*_CTX-M_ of class A (four probes for the identification of *bla*_CTX-M_ subclusters and probes for two SNP positions in each subcluster) and probes for the identification of eight types of carbapenemase genes (*bla*_KPC_ of class A; *bla*_NDM_, *bla*_VIM_, *bla*_IMP_, *bla*_SPM_, *bla*_SIM_, and *bla*_GIM_ of class B; and *bla*_OXA-23_, *bla*_OXA-40_, *bla*_OXA-48_, *bla*_OXA-51_, and *bla*_OXA-58_ of class D). The positive control probe was used to normalize the results of the hybridization. For this purpose, a biotin-labeled oligonucleotide complementary in structure to the PHC probe was added to each hybridization mixture.

The microarrays were tested using DNA samples isolated from well-characterized control strains. The method of hybridization analysis consisted of several steps involving the amplification of target *bla* genes in multiplex PCR with simultaneous labeling with biotin and the subsequent hybridization of amplicons with oligonucleotide probes immobilized in the wells. After hybridization, biotin molecules in the DNA duplexes were stained with the streptavidin–HRP conjugate, followed by colorimetric HRP detection. A typical colorimetric image of the microarray is shown in Figure 4b for the testing of target DNA obtained from the control strain harboring TEM-1, SHV-1, and CTX-M-9 *bla* genes.

Then, different types of *bla* genes of class A (TEM-3, TEM-6, TEM-37, SHV-1, SHV-2, SHV-5, CTX-M-3, CTX-M-9, CTX-M-15, and CTX-M-42) and carbapenemase genes of classes A, B, and D (KPC-3, VIM-1, VIM-2, IMP-1, IMP-2, SPM-1, NDM-1, OXA-23, OXA-24, OXA-48, OXA-51, and OXA-58) were tested separately and in various combinations.

The processing of the staining results was as follows: At the first stage, only significant staining intensities were determined, which differed from the background staining by more than three standard deviations. Then, the relative staining intensities were calculated for identification probes using net staining intensities normalized to the positive control staining value. To determine the SNP, the result of complementary hybridization with the highest signal intensity was considered the perfect match (PM), and the remaining ones with lower signal intensities were regarded as mismatches (MM). The MM values were normalized to the PM values. As a result, the PM values for each probe set in SNP identification equal 1, and the relative intensities of the MMs indicate the degree of non-specific hybridization.

**Figure 5:**
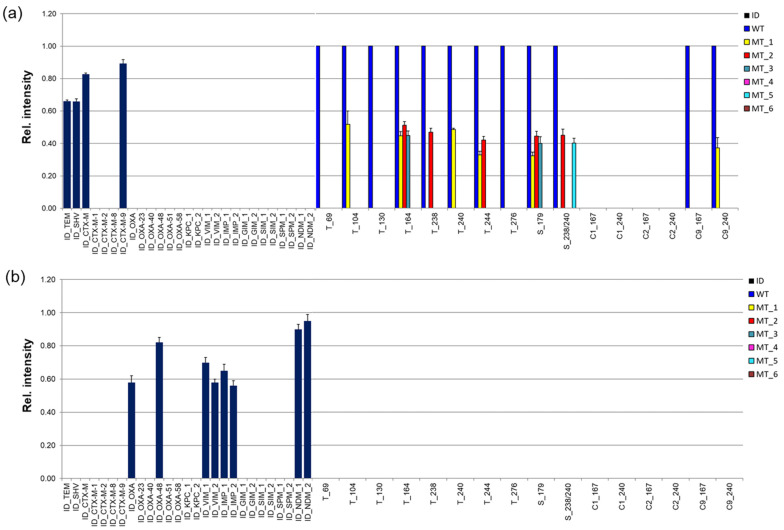
Relative intensities of microarray spot staining after a hybridization experiment with 750 ng of target DNA at 45 °C for 1.0 h. Relative intensities were averaged from three independent hybridizations of (**a**) target DNA obtained from the control strain harboring the TEM-1, SHV-1, and CTX-M-9 β-lactamase genes; (**b**) target DNA obtained from the control strains harboring the VIM-1, IMP-1, NDM-1, and OXA-48 carbapenemase genes. The dark blue color corresponds to the results of determining the type of *bla* gene, the remaining colors correspond to the identification of the SNPs.

Examples of the relative intensity profiles are shown in Figure 5. Figure 5a shows a result of the identification of *bla* genes of a strain harboring TEM-1, SHV-1, and CTX-M-9 genes; Figure 5b shows a result of the identification of a mixture of DNA samples, including VIM-1, IMP-1, NDM-1, and OXA-48 *bla* genes. The values of the mean relative intensity for each oligonucleotide probe were averaged from three independent experiments (they were carried out in different wells of the same plate).

It was shown that the specificity of the β-lactamase and carbapenemase gene identification was high for each DNA target that was being studied. All target DNAs were hybridized both individually and in combinations only with specific probes, while the level of nonspecific hybridization did not exceed the specified interval. All mutation positions in the *bla* genes were identified correctly and without ambiguity. The mean relative MM values varied (from 0 to 0.55), but 90% of the values remained below 0.5.

**Figure 6:**
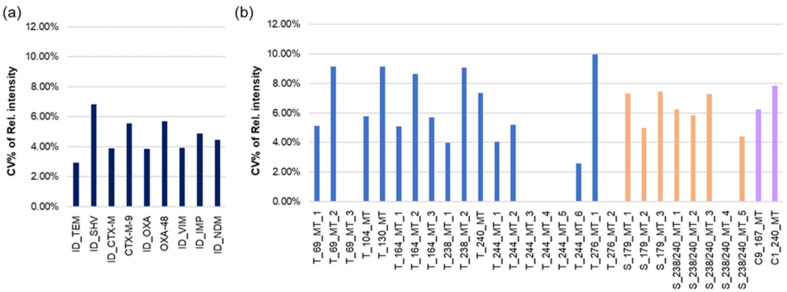
Reproducibility of relative intensities of microarray spot staining after three independent hybridizations in separate wells of the microtiter plate. (**a**) hybridization with the identification probes; (**b**) hybridization with the probes for SNP positions. Different colors correspond to different types of *bla* genes.

An important feature of the hybridization analysis of DNA targets in the microtiter plate is the high reproducibility of the results: the relative standard deviations of the identification probe staining were in the range of 4–6%, and the relative standard deviations for the MMs were in the range of 4–9% (Figure 6). Compared to reproducibility using the same oligonucleotide probes in the microarray on porous membranes [24], these data are noticeably better.

The validation of the microarray was carried out by testing 65 DNA samples isolated from the clinical strains *Enterobacteriaceae*, *Pseudomonas aeruginosa*, and *Acinetobacter baumannii* (Table S1). A total of 9 samples were sensitive to β-lactams and did not harbor any *bla* genes; 6 samples were resistant to penicillines and cephalosporines of I generation only (no ESBL or carbapenemase production); 38 samples were resistant to cephalosporines of III-IV generations (phenotype ESBL+); and 12 samples were resistant to carbapenems (phenotype carbapenemases+).

Table S1 shows the results of the hybridization analysis of these samples on the microarrays in microtiter plates. No false-positive or false-negative results concerning the presence of the *bla* genes were obtained. TEM-1 and SHV-1 penicillinase genes were identified in six samples resistant to penicillins and cephalosporines of the I generation. In 38 samples with the ESBL+ phenotype, individual ESBL genes (SHV or CTX-M type) were detected, as well as combinations of β-lactamase and ESBL genes, including from two to four genes. Twelve samples exhibiting the carbapenemase+ phenotype contained combinations of β-lactamase, ESBL, and carbapenemase genes. The identification of the *bla* genes by microarrays in microtiter plates showed 100% agreement with the data on microbiological phenotyping, RT-PCR, and sequencing. The developed microarray allowed the precise determination of the *bla* gene type and its particular subcluster (for CTX-M and OXA β-lactamases). The microarray also revealed selected SNPs coding for key mutations in class A β-lactamases that expand substrate specificity and resistance to inhibitors.

Therefore, the developed microarray allows for the highly specific identification of selected β-lactamase and carbapenemase genes with the division of individual gene types into subclusters, even for multi-drug resistant strains carrying multiple *bla* genes.

Molecular genotyping by microarrays in microtiter plates is characterized by high performance; 24 samples can be tested in two repetitions on one plate. Incubation and washing can be carried out on automatic devices designed for enzyme immunoassay. The overall time of the sample analysis was no more than 3.5 h, including 1.5 h for DNA amplification and labeling, 1.25 h for hybridization and washing, and 0.75 h for colorimetric detection.

### 3.4. Microarrays in the Wells of the Microtiter Plate for the Quantification of mRNAs of β-Lactamases

The high reproducibility of the multiplex microarray analysis on microtiter plates and the possibility of parallel testing a large number of samples on one plate allow us to develop a quantitative DNA analysis. This task is highly relevant in connection with studying the mechanisms of activation and suppression of *bla* gene expression in bacteria that are resistant to multiple antibiotics.

To determine the concentration of specific mRNA of *bla* genes, the format of DNA analysis on the microarrays in the wells can be used, supplemented by sample preparation, which consists of isolating the RNA transcripts and obtaining cDNA from them in a reaction of reverse transcription. cDNA is then used as a matrix for the amplification of the *bla* genes in multiplex PCR. Next, the target DNA is analyzed on a microarray, as described above.

**Figure 7:**
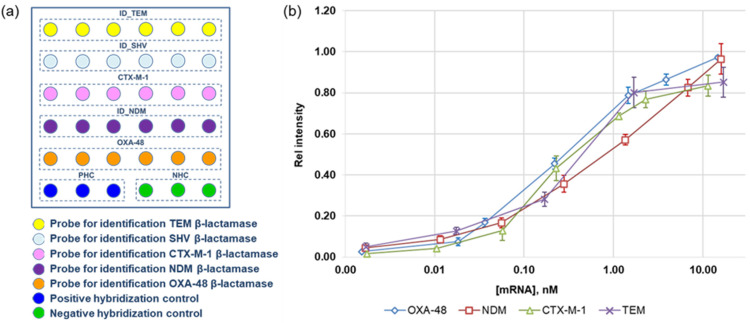
(**a**) The layout of specific and control oligonucleotide probes on a second microarray in the well for quantification of mRNAs. (**b**) Calibration curves for the determination of the mRNA concentration of four types of *bla* genes.

For the multiplex determination of specific mRNA *bla* genes, we designed a second microarray in the well of a microtiter plate (Figure 7a). The microarray included five types of specific oligonucleotide probes for determining clinically significant *bla* genes of different classes (TEM, SHV, and CTX-M of class A, NDM of class B, and OXA-48 of class D). The microarray was prepared in a format of 6 × 6 spots and included five specific oligonucleotide probes for *bla* genes and two control probes. In order to improve the reproducibility of quantification, specific probes were spotted in six repetitions, and control probes were spotted in three repetitions. The total number of spots in each microarray was 36. A positive control probe was used to normalize the results of the hybridization.

For quantification, we suggest using the conventional principle of building calibration curves using standard mRNA samples. PCR and transcription in vitro were utilized to generate mRNA samples of four *bla* genes (TEM-1, CTX-M-1, NDM-1, and OXA-48) using plasmid DNA isolated from *E. coli*, which are the producers of recombinant β-lactamases. Their sequences matched those of the full-sized *bla* genes. Sets of standard mRNA samples of each type, ranging in concentration from 0.001 to 20 nM, were utilized for the microarray testing. Following that, cDNA and biotin-labeled DNA targets were obtained from them through reverse transcription and multiplex PCR procedures. After that, biotinylated DNA targets were hybridized on the microarrays. Both a biotinylated control oligonucleotide and a biotinylated target DNA from a standard sample were present in the hybridization mixture. Each mixture was tested twice (in two separate wells). The staining intensity of each mean probe was normalized to the positive control’s mean staining value. The calibration curves of the relative staining intensities on mRNA concentration were constructed using these data (Figure 7b).

The LODs for each type of mRNA were as follows: 5 ± 0.7 pM (CTX-M-1), 6.0 ± 0.9 pM (NDM-1), 7 ± 1 pM (TEM-1), and 7 ± 1 pM (OXA-48). The relative standard deviation was limited to 15%. The ranges of detected mRNA concentrations for each type of *bla* gene under investigation turned out to be close, which is highly essential for their simultaneous determination.

Thus, we demonstrated, for the first time, that nucleic acid quantification can actually be achieved using colorimetric microarrays in the wells of microtiter plates. The quantitative determination of mRNAs is based on the use of standard mRNA samples, which match the size of the entire *bla* gene and pass all analytical steps together with the sample. The productivity of the method is greatly increased by arranging microarrays in the wells of a microtiter plate. In this work, we determined the concentration of specific mRNAs of four types of *bla* genes. In the future, the number of detected mRNAs can be increased. The use of this approach makes it possible to shift from a semi-quantitative to a quantitative analysis of gene transcripts, which is required when investigating the molecular mechanisms of antibiotic resistance gene induction in bacteria with multiple antibiotic resistance.

## 4. Conclusions

Bacterial resistance to antibiotics is the main factor in therapy’s failure in clinical practice. The emergence and wide spread of multi-antibiotic resistant bacteria has made the development of new multiplexed techniques extremely relevant. This work reports on the development of a novel microarray technique that uses the photoactivation of polystyrene with bifunctional linkers for the covalent attachment of oligonucleotide probes in the wells of a microtiter plate. Taking into consideration the potential of the 96-well format for combining microarrays with various specificities, we first applied this technology to develop microarrays for the identification of genes of the most clinically relevant β-lactamases (ESBLs and inhibitor-resistant β-lactamases (IRT) of class A and carbapenemases of classes A, B, and D). A collection of DNA samples isolated from clinical strains was used to validate the method, and the results showed that it had high specificity for identifying *bla* genes, including those in multicomponent mixtures. The use of plates can significantly increase the number of samples analyzed in parallel, improve the accuracy and reproducibility of the results, and shorten the overall analysis time when automated systems are used. The high reproducibility of the results made it possible to develop, for the first time, a microarray for the quantification of specific β-lactamase mRNAs. A novel microarray was developed in order to simultaneously quantify the mRNA genes of four β-lactamase gene types. The basis for the quantitative determination of mRNAs is the use of standard mRNA samples, which match the size of the entire *bla* genes and pass all analytical steps together with clinical samples. Using 96-well plates allows for the testing of both standard and clinical samples on the same plate. The method is highly sensitive and reproducible. In this work, we determined the concentrations of specific mRNAs of four types of *bla* genes. In future work, the number of detected mRNAs can be increased. The combination of reliable performance, high throughput in conventional 96-well plates, with inexpensive colorimetric detection on flatbed scanners makes the microarrays suitable for routine clinical application and for the study of multi-drug resistant bacteria.

## Data Availability

The research data are stored in the department.

## Supplementary Materials

The following supporting information can be downloaded at https://www.mdpi.com/article/10.3390/cimb46010005/s1, Table S1: The results of genotyping DNA samples isolated from clinical bacterial strains on microarrays in the wells of the microtiter plate.
