# Supplementary material for: Multiplex Microarrays in 96-Well Plates Photoactivated with 4-Azidotetrafluorobenzaldehyde for the Identification and Quantification of β-Lactamase Genes and Their RNA Transcripts
Source: Curr Issues Mol Biol. 2023 Dec 20;46(1):53–66. doi: 10.3390/cimb46010005 (PMC10814224; doi:10.3390/cimb46010005)
Supplement: Supplementary file 1 [file cimb-46-00005-s001.zip › cimb-2762072-supplementary.pdf]

Supplementary Materials

Multiplex Microarrays in 96-Well Plates Photoactivated with 4-Azidotetrafluorobenzaldehyde for the Identification and Quantification of β-Lactamase Genes and Their RNA Transcripts

Mariya M. Ulyashova, Galina V. Presnova, Anna A. Filippova, Vitaly G. Grigorenko, Alexey M. Egorov and Maya Yu. Rubtsova *

Department of Chemistry, Lomonosov Moscow State University, 119991 Moscow, Russia; mmu@enzyme.chem.msu.ru (M.M.U.); gkovba@enzyme.msu.ru (G.V.P.); iiffii@mail.ru (A.A.F.); GrigorenkoVG@my.msu.ru (V.G.G.); aegorov@enz.chem.msu.ru (A.M.E.)

* Correspondence: myr@enz.chem.msu.ru

Table S1. The results of genotyping DNA samples isolated from clinical bacterial strains on microarrays in the wells of the microtiter plate.

BL type	Strain	Microarray genotyping
No BL genes detected (N = 9)	*Escherichia coli (N = 2)*	N/D
	Klebsiella pneumoniae (N = 2)	N/D
	Proteus mirabilis (N = 1)	N/D
	Acinetobacter baumannii (N = 2)	N/D
	Pseudomonas aeruginosa (N = 2)	N/D
Penicillinases (N = 6)	*Escherichia coli (N = 2)*	TEM-1-like (N = 2)
	Klebsiella pneumoniae (N = 2)	SHV-1-like (N = 2)
	Serratia marcescens (N = 2)	TEM-1-like (N = 2)
ESBL(s) (including the presence of penicillinases) (N = 38)	*Escherichia coli (N = 10)*	TEM-1-like + SHV-5-like* (N = 2)
		CTX-M-1-like (N = 1)
		CTX-M-15-like* (N = 1)
		CTX-M-9-like (N = 1)
		TEM-1-like + CTX-M-15-like* (N = 2)
		TEM-1-like + CTX-M-23-like* (N = 1)
		TEM-1-like + CTX-M-9-like (N = 1)
		SHV-5-like* + CTX-M-9-like (N = 1)
	Klebsiella pneumoniae (N = 11)	SHV-1-like + SHV-5-like* (N = 2)
		SHV-1-like + CTX-M-15-like* (N = 1)
		TEM-1-like + SHV-1-like + SHV-2-like* (N = 1)
		TEM-1-like + SHV-1-like + CTX-M-15-like* (N = 2)
		TEM-1-like + SHV-1-like + CTX-M-9-like

BL type	Strain	Microarray genotyping
		(N = 1)
		TEM-1-like + SHV-1-like + CTX-M-16-like* (N = 1)
		TEM-1-like + SHV-1-like+SHV-5-like* + CTX-M-1-like (N = 2)
		TEM-1-like + SHV-1-like+SHV-5-like* + CTX-M-2-like (N = 1)
	Enterobacter cloacae (N = 5)	SHV-5-like* (N = 1)
		CTX-M-1-like (N = 1)
		TEM-1-like + CTX-M-1-like (N = 1)
		TEM-1-like + CTX-M-15-like* (N = 1)
		SHV-5-like + CTX-M-9-like (N = 1)
	Enterobacter aerogenes (N = 1)	TEM-1-like + CTX-M-1-like (N = 1)
	Citrobacter freundii (N = 2)	CTX-M-15-like* (N = 1)
		TEM-1-like + CTX-M-15-like* (N = 1)
	Proteus mirabilis (N = 4)	TEM-1-like + CTX-M-1-like (N = 1)
		TEM-1-like + CTX-M-15-like* (N = 1)
		TEM-1-like + SHV-2-like* + CTX-M-2-like (N = 2)
	Providencia rettgeri (N = 2)	CTX-M-15-like (N = 1)
		TEM-1-like + CTX-M-15-like* (N = 1))
	Morganella morganii (N = 1)	CTX-M-15-like* (N = 1)
	Salmonella infantis (N = 1)	TEM-1-like + CTX-M-15-like* (N = 1)
	Serratia marcescens (N = 1)	CTX-M-1-like
Carbapenemase(s) (including the presence of penicillinases and ESBLs) (N = 12)	*Acinetobacter baumannii (N = 5)*	OXA-51-like + OXA-23-like (N = 1)
		OXA-51-like + OXA-40-like (N = 2)
		OXA-51-like + OXA-58-like (N = 2)
	Pseudomonas aeruginosa (N = 4)	VIM-like (N = 4)
	Klebsiella pneumoniae (N = 5)	SHV-1-like + OXA-48-like (N = 1)
		SHV-1-like + NDM-like (N = 1)
		TEM-1-like + SHV-1-like + OXA-23-like + KPC-like (N = 1)
		TEM-1-like + SHV-1-like + CTX-M-15-like* + NDM-like (N = 1)
		TEM-1-like + SHV-1-like + CTX-M-15-like* + OXA-48-like (N = 1)

*SHV-2-like (GE238/240SE substitution)
SHV-5-like (SHV_ID + GE238/240SK substitution)
CTX-M-15-like (D240G substitution)
CTX-M-23-like (P167T substitution)
CTX-M-16-like (D240G substitution)
